# A Direct Comparison of Local-Global Integration in Autism and other Developmental Disorders: Implications for the Central Coherence Hypothesis

**DOI:** 10.1371/journal.pone.0039351

**Published:** 2012-06-19

**Authors:** Inês Bernardino, Susana Mouga, Joana Almeida, Marieke van Asselen, Guiomar Oliveira, Miguel Castelo-Branco

**Affiliations:** 1 Visual Neuroscience Laboratory, IBILI, Faculty of Medicine, Coimbra, Portugal; 2 Neurodevelopment and Autism Department from Child Center of Pediatric Hospital of Coimbra, Coimbra, Portugal; 3 Research and Clinical Training Department from Pediatric Hospital of Coimbra, Coimbra, Portugal; Queen Mary University of London, United Kingdom

## Abstract

The weak central coherence hypothesis represents one of the current explanatory models in Autism Spectrum Disorders (ASD). Several experimental paradigms based on hierarchical figures have been used to test this controversial account. We addressed this hypothesis by testing central coherence in ASD (n = 19 with intellectual disability and n = 20 without intellectual disability), Williams syndrome (WS, n = 18), matched controls with intellectual disability (n = 20) and chronological age-matched controls (n = 20). We predicted that central coherence should be most impaired in ASD for the weak central coherence account to hold true. An alternative account includes dorsal stream dysfunction which dominates in WS. Central coherence was first measured by requiring subjects to perform local/global preference judgments using hierarchical figures under 6 different experimental settings (memory and perception tasks with 3 distinct geometries with and without local/global manipulations). We replicated these experiments under 4 additional conditions (memory/perception*local/global) in which subjects reported the correct local or global configurations. Finally, we used a visuoconstructive task to measure local/global perceptual interference. WS participants were the most impaired in central coherence whereas ASD participants showed a pattern of coherence loss found in other studies only in four task conditions favoring local analysis but it tended to disappear when matching for intellectual disability. We conclude that abnormal central coherence does not provide a comprehensive explanation of ASD deficits and is more prominent in populations, namely WS, characterized by strongly impaired dorsal stream functioning and other phenotypic traits that contrast with the autistic phenotype. Taken together these findings suggest that other mechanisms such as dorsal stream deficits (largest in WS) may underlie impaired central coherence.

## Introduction

A cognitive theory - the Weak Central Coherence (WCC) account [1] - has been proposed to address cognitive weaknesses and strengths in Autism Spectrum Disorder (ASD). ASD is characterized by a symptomatic triad including severely impaired social interaction, deficits in communication and restricted/stereotyped patterns of behavior, interests and activities [2], [3]. Superior visuospatial skills have been described in ASD, particularly in which concerns visual search [4], [5], [6] and puzzle assembly tasks [7], [8]. Nevertheless, there is some evidence of a distinctive visual perceptual style in this disorder that has been considered to account for high level deficits particularly in the face processing domain [9], [10], [11].

The WCC account describes the perceptual and cognitive biases in ASD according to the claim that these patients perceive visual scenes as a sparse set of details rather than as a congruent and meaningful unit, failing in the extraction of the global configuration [1], [12]. This hypothesis explains the cognitive phenotype of ASD in terms of dissociation between local and global information processing that has been mostly analyzed in the visual domain.

An extensive range of experimental paradigms have been used to measure WCC in ASD, namely the block design subtest [7], [13], the embedded-figure test [14], [15], the copying impossible-figure [16] and visual illusion tasks [17], [18]. However, the main paradigm in this domain has been the study of coherent visual processing using hierarchical figures, such as Navon stimuli [15], [19], [20], [21]. This stimulus type provides an explicit separation of both local and global levels of visual processing. The pattern of findings has been inconsistent suggesting the need for controlled experiments in multiple clinical populations directly testing the main statement of that hypothesis [22], [23], [24], [25], [26]. Plaisted et al. [20] reported this pattern of mixed findings by showing that ASD patients exhibited local advantage and local interference effects in a divided attention task while they showed a global precedence effect (as typically developing participants did) in a selective attention task. The authors explain these differences based on the different task demands by suggesting that there is an intact global processing in ASD patients alongside with a voluntarily selective attention bias to local information in the absence of overt instructions. This is in line with the enhanced perceptual functioning model (EPF) which postulates an autistic perceptual endophenotype characterized by locally-oriented processing in ASD without disrupting global information processing aspects [27], [28]. This model is consistent with the observation that tasks with high perceptual load conduct to superior performance in ASD patients in contrast with typically developing controls [29].

Although the WCC account has been formulated to explain the distinctive cognitive phenotype of ASD, the detailed-focused cognitive style has been described in other developmental disorders, such as Williams syndrome (WS), although a distinct behavioral phenotype is observed in this condition. This leads to a question about the distinctiveness of the WCC in ASD and renders the direct comparison between the ASD and WS phenotypes important for the elucidation of this debate.

WS is a genetic neurodevelopmental disorder characterized by predominant visuospatial impairment [30], [31] contrasting with relatively spared verbal processing [32], [33], [34]. Visual dorsal stream deficits are the hallmark of this disorder [35]. These visuospatial deficits have been explored in terms of local-global visual processing and a local processing bias in this disorder is particularly evident in the visuoconstructive domain [36], [37].

The mixed findings particularly found in ASD emphasize the notion that global processing in these disorders seems to be affected under some task conditions and spared under others. Therefore, it is crucial to test the central coherence abilities in ASD (and other neurodevelopmental conditions) under the same task requirements in order to clarify their pattern of visual processing. In this domain, there are several important methodological issues that should be addressed to better understand the pattern of visual perception of these developmental disorders. Accordingly, it is important to separate the ability to perceive global information from the detailed focused cognitive style characterized by the preferential use of local approaches when analyzing a visual scene in the absence of overt instructions. Indeed, some studies focused on attention tasks giving direct instructions to attend to either local or global levels of information [16], [20], [36] while others only required free viewing to assess preference [21], [37]. So far, this crucial distinction remains to be done, in the same study, under the same task conditions, and with different clinical populations and control groups. In the current study, we explicitly separated perceptual bias and cognitive style from global processing impairment by using multiple measures of central coherence based on the classical Navon paradigm [19]. In addition, we also attempted a separation between perceptual and visuoconstructive components of local-global processing, which has not been addressed before in ASD (in contrast with WS).

**Table 1:** Characteristics of clinical and control groups.

	Chronological Age (years)		Education (years)		IQ (WISC-III or WAIS-III)		Gender
	Mean (SE)	Range	Mean (SE)	Range	Mean (SE)	Range	(m:f)
**WS (n = 18)**	17.33 (1.81)	8–34	5.00 (1.00)	0–12	53.94 (2.01)	42–75	11∶7
**ASD_ID (n = 19)**	13.26 (0.58)	10–18	6.74 (0.43)	4–9	64.47 (1.76)	52–79	15∶4
**ASD_noID (n = 20)**	12.10 (0.46)	10–17	6.55 (0.48)	4–11	103.40 (2.41)	90–129	20∶0
**C_TD (n = 20)**	15.70 (1.81)	7–34	6.80 (0.79)	2–14	107.94 (1.73)	95–119	11∶9
**C_ID (n = 20)**	15.35 (0.96)	10–29	7.15 (0.46)	3–9	58.60 (1.95)	40–74	15∶5

Note. WS  =  Williams Syndrome group; ASD_ID  =  Autism Spectrum Disorders group with intellectual disability; ASD_noID  =  Autism Spectrum Disorders group without intellectual disability; C_TD  =  typically developing control group; C_ID  =  control group with intellectual disability; WISC-III  =  Wechsler Intelligence Scale for Children, 3^rd^. ed.; WAIS-III  =  Wechsler Adult Intelligence Scale, 3^rd^ ed.

In sum, the WCC account proposes that detailed-focused cognitive style in ASD is a contributory cause of some characteristics of this disorder, in particular defective face processing [10]. However, the fact that WCC is a common denominator of both ASD and WS is at odds with their substantially distinct cognitive profile. Therefore, we predict that if WCC is distinctive in ASD and underlies its pathophysiology the coherence deficits should dominate in ASD patients. Although we believe that such deficits may emerge under certain conditions (as also found in this study), this would also mean that they do not provide a full account of the phenotype and that complementary mechanisms, such as dorsal stream deficits, should also be considered. Our main goal was to test the hypothesis that WCC is unique and distinctive to ASD by using classical paradigms of central coherence in clinical populations with clear categorical differences concerning intellectual disability and cognitive phenotype. This would allow us to understand if these conditions share the same underlying cognitive mechanisms of integration or if this perceptual feature can alternatively be considered specific to the pathophysiology of ASD. The understanding of the neurobehavioral relevance of central coherence in ASD requires addressing both perceptual bias and performance levels as well as the impact of intellectual disability in central coherence measures. In the current study, intellectual disability was controlled for by selecting appropriate matched clinical and control groups. Participants were tested under three experimental tasks: a Preference task without a priori “correct” response to assess spontaneous visual processing preferences, a Correct Choice task to evaluate the accuracy in perceiving both global and local information and a Drawing task to explore visuoconstructive integrative abilities. We further explored the effect of the physical presence of the stimulus on the visual processing of hierarchical stimuli by introducing both perceptual and memory conditions. Furthermore, in the preference task, we addressed the invariance of the processing bias to local and global rotation manipulations that were introduced to increase task sensitivity in the detection of mild perceptual bias.

## Methods

### Ethics Statement

This study and all the procedures were reviewed and approved by the Ethics Commissions of the Faculty of Medicine of the University of Coimbra (Comissão de Ética da Faculdade de Medicina da Universidade de Coimbra) and of the Pediatric Hospital of Coimbra (Comissão de Ética do Hospital Pediátrico de Coimbra) and was conducted in accordance with the declaration of Helsinki. Written informed consent was obtained from participants older than 18 years of age and from the parents/guardians in the case of participants younger than 18 years of age. Children and adolescents younger than 18 years of age gave oral informed consent.

### Participants

Ninety-seven participants were included in this study: 18 WS patients, 19 ASD patients with intellectual disability (ASD_ID) (Intelligence Quotient (IQ) <80), 20 ASD patients without intellectual disability (ASD_noID) (IQ ≥90), 20 typically developing participants matched for chronological age (C_TD) and 20 control participants with intellectual disability matched for IQ (C_ID). The characteristics of clinical and control groups are summarized in Table 1.

**Figure 1:**
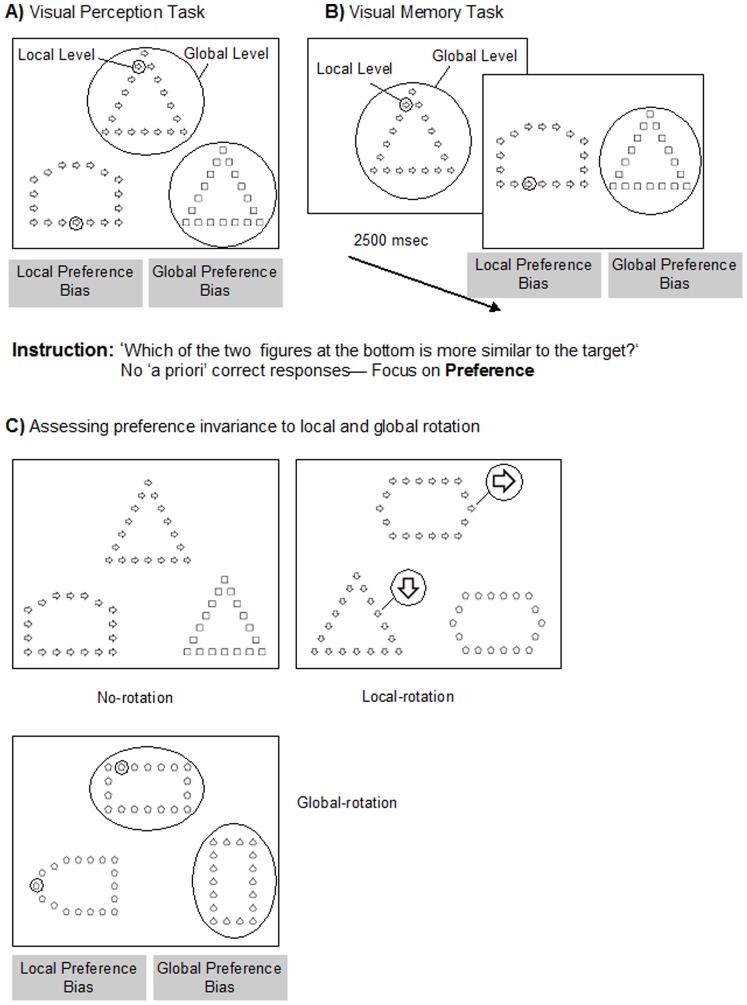
Illustration of the Visual Preference Tasks. Example of the configurations used in A) visual perception preference tasks and B) visual memory preference tasks. C) Illustration of the non-inversion, local-inversion and global-inversion conditions used on visual perception preference and visual memory preference tasks to assess preference invariance to global and local rotation. Note. Figures are presented according to the real scale (not real size) and, therefore, visibility was higher in the experimental task.

ASD participants were recruited from the Neurodevelopment and Autism Department from the Child Center of Pediatric Hospital of Coimbra. ASD diagnoses were assigned on the basis of gold standard instruments such as: parental or caregiver interview (Autism Diagnostic Interview– Revised, ADI-R [38]), direct structured proband assessment (Autism Diagnostic Observation Schedule, ADOS [39]), and clinical examination performed by an experienced neurodevelopmental Pediatrician, based on the diagnostic criteria for autistic disorder from Diagnostic and Statistical Manual of Mental Disorders IV, DSM-IV-TR [2]. All ASD patients had positive results in the ADI-R and ADOS for autism or ASD, and met the criteria for autistic disorder from the DSM-IV-TR. Only idiopathic cases were included (negative kariotypic results in, fluorescence in situ hybridization - FISH ch 15 q11-13 - and FMR1 mutation). In this group, 7 patients were medicated with Risperidone and 2 were medicated with Methylphenidate. However, parents of these children were requested not to give their children the medication on the days of the testing.

WS participants were recruited from a database used in previous studies [30], [31]. All patients were diagnosed based on clinical and genetic examinations confirmed by FISH analysis, which demonstrated the hemyzigous Elastin deletion. Additional genetic analysis sequenced the breakpoint regions and revealed the same deletion size (∼1.55 Mb) in all WS participants.

Control participants matched for IQ were recruited from the same department and from local special education institutes. None of these participants were taking selective serotonin reuptake inhibitor or neuroleptic medications. Co-morbid conditions were explicitly excluded (epilepsy, brain injury, sensory deficits, associated genetic syndromes, and motor deficits that could interfere with task response).

Control participants matched for chronological age were healthy, with no history of psychiatric, neurologic and ophthalmologic illnesses and naïve concerning to the testing procedures. They were recruited from local schools and were individually tested at their own schools.

The parents of participants included in WS and C_ID groups completed the Social Communication Questionnaire to exclude co-morbidity with ASD [40]. The scores were below 15, which is the positive cut-off for ASD. All participants included in the study received the Portuguese adapted version of the Wechsler Intelligence Scale for Children – 3^rd^ edition (WISC-III) [41] or the Wechsler Adult Intelligence Scale – 3^rd^ edition (WAIS-III) [42], according to the participant’s age. The ASD_ID group only includes subjects with IQ inferior to 80 while the ASD_noID group includes subjects with IQ superior or equal to 90, which is consistent with Wechsler definition of intellectual disability [41], [42].

The three clinical groups (WS, ASD_ID and ASD_noID) were matched for chronological age and education level with both C_TD-matched (Mann-Whitney test, *p*>0.05) and C_ID-matched (Mann-Whitney test, *p*>0.05) control groups. Additionally, the clinical groups with intellectual disability (WS and ASD_ID) were matched for IQ (Mann-Whitney test, *p*>0.05) with the C_ID-matched control group.

**Figure 2:**
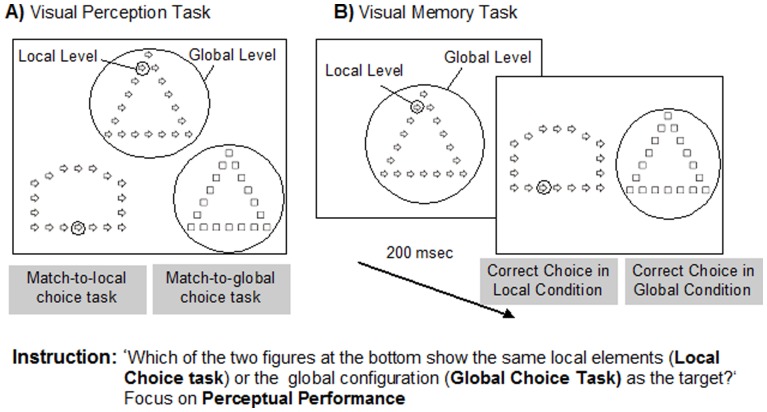
Illustration of the Correct Choice Tasks. Example of the configuration used in A) visual perception correct choice tasks and B) visual memory correct choice tasks. Note. Figures are presented according to the real scale (not real size) and, therefore, visibility was higher in the experimental task. Note that questions posed to participants were in simple Portuguese.

### Procedure

We used Navon’s hierarchical stimuli [19], which consisted of global geometrical figures made up of 18 smaller geometrical figures. In each hierarchical form, the shape of the local level differed from the shape of the global level. The stimuli were shown on a 33,8 cm×27,1 cm computer screen (1280×1024 pixels) using the software package Presentation (Neurobehavioral systems). The size of the local shapes was 0.57° horizontally and 0.57° vertically and the distance between them was 0.57°. The horizontal and vertical sizes of the global shapes differed accordingly to the figure configuration. The color of the stimuli was black and they were shown on a white background at high contrast (95%).

Participants were individually tested in a quiet and darkened room, seated at a distance of 50 cm from the computer screen. They were asked to perform three experimental tasks: a Preference task, a Correct Choice task and, finally, a Drawing task.

#### PreferenceTasks

On each trial, participants performed a match to sample similarity task by comparing two figures with one target figure. This task was performed under different task conditions, in which task requirements (visual perception and visual memory tasks) and the geometric configuration of the stimuli (non-inversion, local-inversion, and global-inversion conditions) were manipulated. For the visual perception preference task, the participants viewed a display containing a target figure at the top of the screen and two comparison figures at the bottom (Figure 1A). One of the comparison figures shared only the global shape with the target figure and the other had the same local elements as the target but had different global configuration. That is, each comparison figure shared only one level (local or global) with the target figure and appeared randomly and equally often on the left and right positions. For the visual memory preference task, each trial comprehended a presentation phase, in which the target figure was shown during 2500 ms, followed by the appearance, without delay, of the two comparison figures (Figure 1B). For both perceptual judgment and memory tasks, participants were asked to indicate which of the two bottom figures was more similar to the target, thereby reporting their visual processing preferences (bias). The instructions for the perceptual preference task were as follows (translated from Portuguese): “In this screen, you have three figures, one up here (pointing) and two below (pointing). You should look closely at all these pictures and indicate, in your opinion, which of the two figures down here (pointing), is more similar to the figure above”. It is important to note that, in this task, there is no correct response and the subjects’ answers reflect only the preferred pattern of visual analysis when analyzing a hierarchical figure.

**Figure 3:**
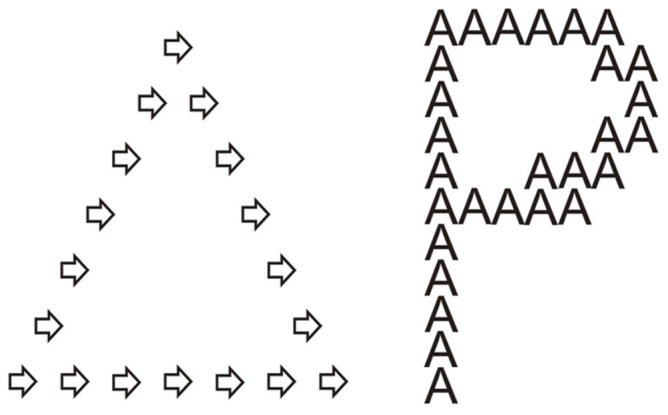
Stimuli used in the drawing task. A simple geometric figure and a letter used in the drawing task.

In both perception and visual memory preference tasks, we included a no-rotation condition in which the local and global information of the comparison figures were presented in the same orientation of the target figure. Additionally, two control conditions (with different geometrical configurations) were also administered, namely local-rotation and global-rotation conditions, in which the orientation of either the local or global elements of the stimulus was manipulated to provide generalization and further, enhance the likelihood of detecting subtle forms of perceptual bias (Figure 1C). The local elements or the global shape were rotated 90 or 180 degrees to ensure that the figures exhibited different orientations of those presented in the target figure. In the local-rotation condition we rotated the local elements of the comparison figure matched for local level with the target figure. This approach favoured a change to a more global bias. In the global-rotation condition, we rotated the global shape of the comparison figure matched for global configuration with the target figure in order to explicitly increase the local similarity. This strategy favoured a change to a local bias.

Participant underwent 20 test trials in each task condition performing a total of 60 test trials. Eight familiarization trials were administered for each task. The familiarization phase was repeated whenever the subjects did not understand the instructions or had difficulties coordinating the motor response. All participants included in the task understood the task instructions. The visual perception task was provided before the visual memory task for all participants.

#### Correct choice tasks

Two different task conditions were included, namely a match-to-local choice task and a match-to-global choice task, differing only on the instruction given to the participants (but both requiring a correct response, unlike the Preference Tasks). In the match-to-local choice task, participants indicated which of the two comparison figures had the same local shapes as the target, while in the match-to-global choice task participants indicated which of the two comparison figures was matched with the target in terms of the global configuration. Additionally, as occurred in the Preference task, participants were performed visual judgments under visual perception and visual memory conditions. In the visual perception correct choice task, participants viewed a display containing one target figure and two comparison figures (Figure 2A). In the visual memory correct choice task, the target figure was presented during 200 ms, followed by the appearance of two comparison figures (Figure 2B). For both experimental tasks, we presented six blocks of eight trials each, alternating between match-to-local and match–to-global conditions (three blocks for each condition). Participants performed a total of 48 trials for each perception and memory conditions. Five consecutive correct practice trials were administered for all conditions to ensure that all participants understood the task instructions.

#### Drawing task

A Drawing (visuoconstructive) task was included, in which participants copied two hierarchical figures (a large triangle made of smaller arrows and a large ‘P’ made of smaller ‘A’s’) (Figure 3). Designs were shown in an A5 paper until participants finished the copy. There was no time limit for completion of the task. A rating scale, similar to that used by Porter & Coltheart [23], was created to rate visuoconstructive integrative ability. Three scores were carried out for each drawing task, namely a local score, a global score and an integration score. For local and global scores, ratings were between 0 (“totally absent”) and 3 (“perfect reproduction”). We computed the local and global scores for each participant by summing the score of the two drawings produced by each participant. In sum, local and global scores had a minimum score of zero and a maximum of six. For the integration score, ratings were 0 (if the local and global shapes were drawn independently) or 1 (if the local and global configurations were accurately integrated as a whole). Two WS participants were not able to draw the triangle and one refused to draw the hierarchical letter resulting in a total of 191 drawings produced by the clinical and control groups which were rated by two independent raters. The raters were not aware that the drawings had been produced by different groups. Inter-rater reliability scores were 0.912 for local score, 0.880 for global score and 0.878 for integration score (Spearman’s rho correlations, *p*<0.05).

### Statistical Analysis

Nonparametric statistics (Mann-Whitney U tests, Fisher’s Exact Tests and Spearman’s Rho correlations) were carried out for all statistical analyses to avoid biases due to deviations from normality and variance heterogeneity. All statistical analyses were performed with the IBM SPSS Statistics 19.0 software package.

## Results

### Preference (bias) Tasks

**Table 2:** Group comparison analyses for Preference Tasks considering visual perception and visual memory preference tasks as well as no-rotation, local-rotation and global-rotation task conditions.

	Visual Perception Preference Task			Visual Memory Preference Task		
	No-rotation	Local-rotation	Global-rotation	No-rotation	Local-rotation	Global-rotation
ASD_ID vs. C_TD	p = 0.080	p = 0.623	p = 0.715	p = 0.112	p = 0.122	p = 0.013*
ASD_ID vs. C_ID	p = 0.852	p = 0.314	p = 0.143	p = 0.763	p = 0.852	p = 0.550
ASD_noID vs. C_TD	p = 0.585	p = 0.988	p = 0.322	p = 0.483	p = 0.797	p = 0.560
WS_local vs. C_TD	p = 0.000**	p = 0.001*	p = 0.002*	p = 0.001*	p = 0.001*	p = 0.035*
WS_local vs. C_ID	p = 0.002*	p = 0.003*	p = 0.002*	p = 0.000**	p = 0.003*	p = 0.090
WS_global vs. C_TD	p = 0.018*	p = 0.182	p = 0.087	p = 0.006*	p = 0.022*	p = 0.009*
WS_global vs. C_ID	p = 0.541	p = 0.915	p = 0.884	p = 0.149	p = 0.586	p = 0.487

All comparisons signaled in red are significant and related to increased local bias.

Mann-Whitney U tests; * p<0.05, **p<0.001.

WS_local  =  Williams Syndrome subgroup with local bias; WS_global  =  Williams Syndrome subgroup with global bias; ASD_ID  =  Autism Spectrum Disorders group with intellectual disability; ASD_noID  =  Autism Spectrum Disorders group without intellectual disability; C_TD  =  typically developing control group; C_ID  =  control group with intellectual disability.

**Figure 4:**
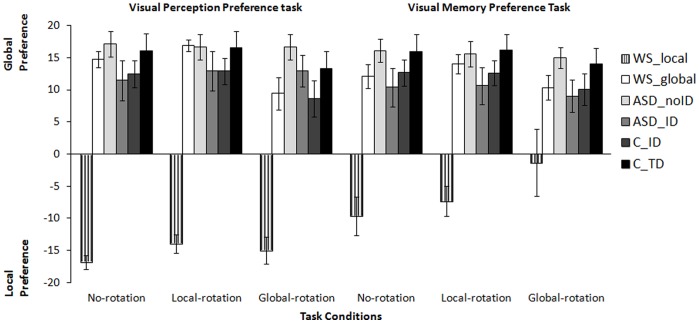
Mean percentage of global responses for all clinical and control groups for the visual perception preference task conditions and the visual memory preference task conditions. Given the bimodal pattern found in WS only for this task, and for sake of clarity we plot two WS subgroups, according to dominantly local or global preference (see text). WS_local  =  Williams Syndrome subgroup with local bias; WS_global  =  Williams Syndrome subgroup with global bias; ASD_ID  =  Autism Spectrum Disorders group with intellectual disability; ASD_noID  =  Autism Spectrum Disorders group without intellectual disability; C_TD  =  typically developing control group; C_ID  =  control group with intellectual disability.

**Table 3:** Group comparison analyses for Correct Choice Tasks considering visual perception and visual memory correct choice tasks as well as match-to-global and match-to-local task conditions.

	Visual Perception Correct Choice Task		Visual Memory Correct Choice Task	
	Global condition	Local condition	Global condition	Local condition
ASD_ID vs. C_TD	p = 0.012*	p = 0.131	p = 0.020*	p = 0.012*
ASD_ID vs. C_ID	p = 0.479	p = 0.162	p = 0.282	p = 0.283
ASD_noID vs. C_TD	p = 0.223	p = 0.171	p = 0.452	p = 0.659
WS vs. C_TD	p = 0.002*	p = 0.000**	p = 0.000**	p = 0.000**
WS vs. C_ID	p = 0.158	p = 0.024*	p = 0.001*	p = 0.037*

All comparisons signaled in red are significant and related to increased number of errors.

Mann-Whitney U tests; * p<0.05, **p<0.001.

WS  =  Williams Syndrome group; ASD_ID  =  Autism Spectrum Disorders group with intellectual disability; ASD_noID  =  Autism Spectrum Disorders group without intellectual disability; C_TD  =  typically developing control group; C_ID  =  control group with intellectual disability.

**Figure 5:**
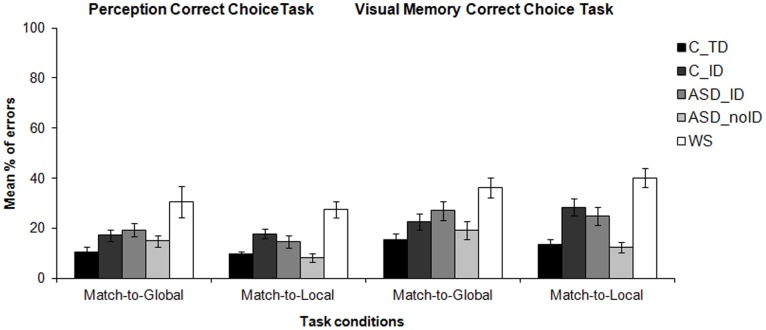
Mean percentage of errors for all clinical and control groups for the visual perception and visual memory “correct choice” task conditions. WS  =  Williams Syndrome group; ASD_ID  =  Autism Spectrum Disorders group with intellectual disability; ASD_noID  =  Autism Spectrum Disorders group without intellectual disability; C_TD  =  typically developing control group; C_ID  =  control group with intellectual disability.

#### Visual perception preference task

Group analyses revealed that both ASD clinical groups with or without intellectual disability (ASD_ID and ASD_noID) have a relative preference for global configurations in all no-rotation, local-rotation and global-rotation conditions. Surprisingly, their choice behavior was similar to both C_TD and C_ID control groups. Thus, no significant differences were found between the ASD clinical groups and respective control groups in all task conditions (Mann-Whitney U test, *p*>0.05, see Table 2 for details on exact p-values and specific comparisons).

Conversely, in the WS group we found a bimodal distribution specifically for this task, with a subgroup showing a clear preference for local strategies (with more than 80% of local choices) while the other subgroup showed a clear global visual bias (with more than 80% of global choices). The WS subgroup who preferred local bias showed significantly more local choices than both C_TD and C_ID controls groups on all task conditions (Mann-Whitney U test, *p*<0.05). Concerning the WS subgroup who preferred global choices, no significant differences were found when comparing with the C_ID control group for all task conditions (Mann-Whitney U test, *p*>0.05), however, significant differences were found when comparing with the C_TD group but only for the local-rotation condition (Mann-Whitney U test, *p*<0.05) and the global-rotation condition (Mann-Whitney U test, *p*<0.05).

**Table 4:** Comparison of blinded Local and Global scores obtained from two independent raters of the visuoconstructive drawing task.

	Drawing Task	
	Global score	Local score
ASD_ID vs. C_TD	p = 0.092	p = 0.051
ASD_ID vs. C_ID	p = 0.567	p = 0.396
ASD_noID vs. C_TD	p = 0.721	p = 0.222
WS vs. C_TD	p = 0.000**	p = 0.000**
WS vs. C_ID	p = 0.000**	p = 0.000**

All comparisons signaled in red are significant and related to lower global and local scores.

Mann-Whitney U tests; **p<0.001.

WS  =  Williams Syndrome group; ASD_ID  =  Autism Spectrum Disorders group with intellectual disability; ASD_noID  =  Autism Spectrum Disorders group without intellectual disability; C_TD  =  typically developing control group; C_ID  =  control group with intellectual disability.

**Figure 6:**
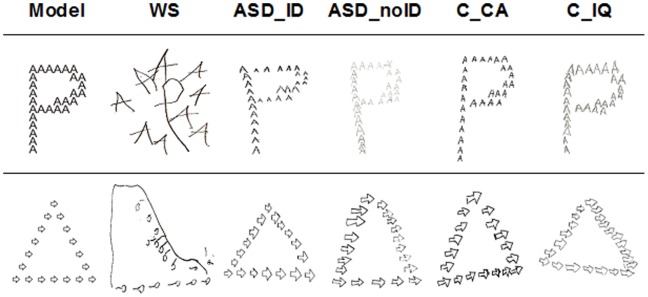
Examples of drawings produced by clinical and control groups. WS  =  Williams Syndrome group; ASD_ID  =  Autism Spectrum Disorders group with intellectual disability; ASD_noID  =  Autism Spectrum Disorders group without intellectual disability; C_TD  =  typically developing control group; C_ID  =  control group with intellectual disability.

**Table 5:** Comparison of blinded Integration score obtained from two independent raters.

	Drawing Task: Integration Score	
	‘P’ Drawing	Triangle Drawing
ASD_ID vs. C_TD	p = 0.106	p = 0.155
ASD_ID vs. C_ID	p = 0.283	p = 0.305
ASD_noID vs. C_TD	p = 0.500	p = 0.500
WS vs. C_TD	p = 0.000**	p = 0.004*
WS vs. C_ID	p = 0.000**	p = 0.001*

All comparisons signaled in red are significant and related to lower integration scores.

Fisher’s Exact Test; * p<0.05, **p<0.001.

WS  =  Williams Syndrome group; ASD_ID  =  Autism Spectrum Disorders group with intellectual disability; ASD_noID  =  Autism Spectrum Disorders group without intellectual disability; C_TD  =  typically developing control group; C_ID  =  control group with intellectual disability.

**Figure 7:**
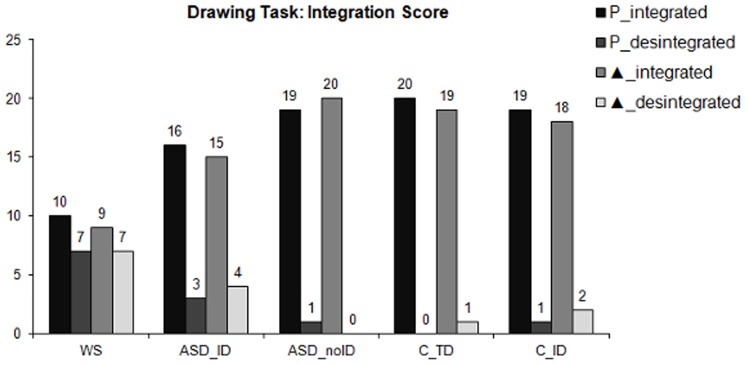
Visuoconstructive integrative abilities. Integration score for all groups indicating the number of subjects who were able to integrate the local elements in order to correctly reproduce the global configuration regarding the geometric hierarchical figure (Triangle) and the hierarchical letter (‘P’). WS  =  Williams Syndrome group; ASD_ID  =  Autism Spectrum Disorders group with intellectual disability; ASD_noID  =  Autism Spectrum Disorders group without intellectual disability; C_TD  =  typical developing control group; C_ID  =  control group with intellectual disability.

#### Visual memory preference task

Similar results were found for the visual memory preference task with group analyses revealing no significant differences between the ASD_ID group and the C_ID across no-rotation, local-rotation and global-rotation conditions (Mann-Whitney U test, *p*>0.05, for details on exact p-values see Table 2). When comparing the ASD_ID group with the C_TD group no significant differences were found for no-rotation (Mann-Whitney U test, *p*>0.05) and local-rotation (Mann-Whitney U test, *p*>0.05) conditions but significant differences emerged for the global-condition (Mann-Whitney U test, *p*<0.05), as expected from the fact that global stimulus rotation induces a local bias. Likewise, no significant differences were found between the ASD_noID and the C_TD (Mann-Whitney U test, *p*>0.005) group, both evidencing a preference for using global strategies when analyzing hierarchical geometric figures irrespective of the control manipulations introduced in the task. In the WS group we replicated the bimodal pattern found in the perception preference task. Significant differences were found between the WS subgroup who preferred local choices and both C_TD and C_ID control groups for all task conditions (Mann-Whitney U test, *p*<0.05), showing a clear preference for using local strategies when performing a match to sample similarity task with no a priori correct responses. Concerning the WS subgroup who preferred global choices, significant differences were found when comparing with the C_TD control group for all task conditions (Mann-Whitney U test, *p*<0.05) but no significant differences were found when comparing with the C_ID control group for all task conditions (Mann-Whitney U test, *p*>0.05). Results are summarized in Table 2 and Figure 4.

### Correct Choice (performance) Tasks

#### Visual perception correct choice task

Group analyses revealed no significant differences when comparing the ASD_ID group with the C_ID group in both match-to-local and match-to-global conditions (Mann-Whitney U tests; *p*>0.05; see Table 3 for details on exact p-values and specific comparisons). However, when comparing the ASD_ID and the C_TD groups, results indicated that the clinical group made significantly more errors than the control group for the match-to-global condition (Mann-Whitney U test; *p*<0.05), but not for the local-to-match condition (Mann-Whitney U test; *p*>0.05). No significant differences were found when comparing ASD_noID group with the matched C_TD control group concerning the identification of local and global similarities (Mann-Whitney U tests; *p*>0.05). Significant differences were found when comparing WS group with the C_TD group in all conditions (Mann-Whitney U tests; *p*<0.05), but when comparing with the C_ID group significant differences were specifically found for the local condition (Mann-Whitney U tests; *p*<0.05).

#### Visual memory correct choice task

Similar results were found as in the perception task. The ASD_ID group performed in a similar way as the C_ID group in all task conditions (Mann-Whitney U tests; *p*>0.05; see Table 3 for further details), but made significantly more errors than the (non IQ-matched) C_TD group on both match-to-local (Mann-Whitney U test; *p*<0.05) and match-to-global (Mann-Whitney U tests; *p*<0.05) task conditions. The WS group made significantly more errors than both C_TD and C_ID control groups in all conditions (Mann-Whitney U tests; *p*<0.05). Results are summarized in Table 3 and Figure 5.

### Visuoconstructive Task Requiring Integration of Local and Global Elements

#### Drawing task

For global and local scores, between-group comparisons revealed that both ASD groups (ASD_ID and ASD_noID) did not differ significantly from the C_TD and the C_ID control groups on both local and global scores (Mann-Whitney U tests; *p*>0.05; see Table 4 for details on exact p-values). Therefore, the ASD groups were able to draw the global and local configuration in a similar way when compared with control participants (see examples in Figure 6). Conversely, significant differences were found when comparing WS group with both control groups regarding local and global scores, which indicates that WS participants were worse at copying global and local shapes (Mann-Whitney U tests; *p*<0.001).

Concerning the integration score, ASD participants as well as their matched control groups were able to integrate the local elements in order to correctly construct the global configuration. Thus, group comparison analyses revealed that the number of subjects who were able to integrate both triangle and ‘P’ drawing did not differ between the ASD groups and the control participants (Fisher’s Exact Test; *p*>0.05; see Table 5 for details on exact p-values). However, considering the WS group, results indicated that they were significantly worse at integrating local and global levels of analysis when compared with C_TD and C_ID control groups (Fisher’s Exact Test; *p*<0.05). Results are summarized in Figure 7.

## Discussion

In this study we tested the hypothesis that WCC is unique and distinctive to ASD. In order to assess this model we used classical markers of central coherence under distinct task constraints in several clinical populations (with categorical distinctions in intellectual disability and cognitive phenotype). Tasks were performed in the perceptual, memory and visuoconstructive domains, with explicit manipulations of levels of bias to better understand the distinction between cognitive style and performance.

ASD participants showed a surprising global preference pattern that is at odds with previous claims [1], although we also replicated local preference under particular conditions (see below). On the other hand, a significant bias towards local information was in general found in the WS group (a model of dorsal stream dysfunction), regardless of IQ. In other words, weakest central coherence was not found in the autistic group but in the WS (with explicitly excluded autistic co-morbidity). Therefore, we found a gradient of central coherence impairment (WS>ASD>C_ID = C_TD) that is not consistent with the pattern derived from the WCC account (ASD>WS>C_ID = C_TD). Interestingly we could experimentally manipulate the preference level, in agreement with the task dependence and clinical heterogeneity found in previous studies [43].

Our study demonstrated that the global bias in ASD patients is accompanied by the presence of a global visual processing adequate to their intellectual level. Conversely, in WS the local bias co-exists with a deficit in correctly perceiving local and global visual information and with clear visuoconstructive integration impairment. It is important to note that ASD patients also showed tendency towards a local bias when experimental manipulations emphasized local processing as occurred in the global-rotation condition in the preference task. In other words, we could replicate the local pattern found in other studies, showing that it can indeed emerge under particular conditions, but that it is not general. Physical properties of the hierarchical stimulus have been described to influence the pattern of global-local processing [44]. Although there is evidence that ASD patients are not vulnerable to changes in visual angle and exposure time [28], it is known that perceptual sensitivity of ASD patients can be modulated by the level of the perceptual task load [29]. The manipulation of levels of stimulus rotation, in our task, may have contributed to the increase of local processing in ASD under these conditions. Moreover, ASD patients were, in general, able to process global information when the level of intellectual disability was controlled for, which agrees with previous claims [13], [15], [24], [25].

Thus, ASD patients may oscillate between a local versus a global mode depending on task requirements and stimulus configuration. A different pattern was detected in WS with consistent local perceptual bias irrespective of task manipulations. Therefore, our findings provide a novel perspective on the WCC debate, without disputing previous findings.

The presence of a stronger detailed-focused perception as well as pronounced coherence deficits in WS may suggest a determinant link between weak central coherence and specific deficits within the dorsal visual stream. WS has been widely referred as involving deficits in tasks subserved by the visual dorsal stream (motion, 2D/3D object coherence and visuoconstructive ability), such as in perceiving 2D form-from-motion stimuli [45], discriminating 2D coherent motion [46] and visuomotor planning [47]. Additionally, Mendes et al. [30] found a 3D coherence deficit, larger than the 2D deficit suggesting that dorsal stream coherence deficits build up in the processing hierarchy. Accordingly, WS patients exhibit a considerable visual coherence and visuoconstructive impairment in particular when they are required to integrate local and global information, which was confirmed by our results. These findings are consistent with identified anatomical abnormalities in the superior parietal sulcus [48], and functional neuroimaging data [49], [50].

Deficits along the dorsal visual stream have also been suggested in ASD, although the results indicate subtle [51], [52] or even inexistent [53], [54] general dorsal stream impairment. This led to the prediction that if central coherence is subserved by dorsal stream processing then it should be weaker in WS than ASD. Our results support this notion. In accordance with this prediction, recently, Poirel et al. [55], studied the shift from local to global visual processing in 6 year-old children and found that the global visual processing is associated to the loss of grey matter in areas along the dorsal visual stream (occipital and parietal visuospatial areas).

In sum, we conclude that abnormal central coherence is not a unique and distinctive characteristic in ASD but may be a marker of dorsal stream dysfunction. Largest dorsal stream deficits are present in populations (WS) with most impaired central coherence and with phenotypic traits (such as hypersociability) that contrast with the autistic phenotype.
